# “Please tell me what happened”: A descriptive study on prevalence, disclosure and characteristics of victimization in people with a psychotic disorder

**DOI:** 10.1371/journal.pone.0219056

**Published:** 2019-07-18

**Authors:** Bertine de Vries, Gerdina H. M. Pijnenborg, Elisabeth C. D. van der Stouwe, Ellen Visser, Steven de Jong, Pharmaco therapy and outcome survey (PHAMOUS)-investigators, Agna A. Bartels-Velthuis, Richard Bruggeman, Stynke Castelein, Frederike Jörg, Wim Veling, André Aleman, Jooske T. van Busschbach

**Affiliations:** 1 University of Groningen, Department of Clinical Psychology, Groningen, the Netherlands; 2 GGZ Drenthe, Department of Psychotic Disorders, Assen, the Netherlands; 3 University of Groningen, University Medical Center Groningen, University Center for Psychiatry, Rob Giel Research Center, Groningen, the Netherlands; 4 University of Groningen, University Medical Center Groningen, Department of Neuroscience, Groningen, the Netherlands; 5 Lentis Psychiatric Institute, Groningen, the Netherlands; 6 GGZ Friesland Mental Health Institution, Leeuwarden, the Netherlands; 7 University of Groningen, University Medical Center Groningen, University Center for Psychiatry, Psychosis Department, Groningen, the Netherlands; 8 Windesheim University of Applied Sciences, Department of Human Movement and Education, Zwolle, the Netherlands; Department of Psychiatry and Neuropsychology, Maastricht University Medical Center, NETHERLANDS

## Abstract

**Introduction:**

Although people with a psychotic disorder are approximately four to six times more often victimized than the general population, victimization is not routinely assessed in mental healthcare. This study investigates prevalence, context and risk factors of victimization in patients with a psychotic disorder in the Northern, relatively rural region of the Netherlands. Moreover, disclosure rates and awareness of psychiatrists are examined.

**Method:**

Information on personal crime (threats, assaults and sexual violence), property and other forms of crime, the context of victimization and disclosure was routinely assessed in 353 patients with a psychotic disorder who received care at a mental health facility. In addition, involved psychiatrists reported on last year’s victimization incidents in their patients.

**Results:**

One third of the patients reported victimization in the previous year. More than half of the crimes were committed by someone acquainted and took place in the victim’s own home or a place familiar to the victim. Younger age, having a comorbid disorder, drug use and perpetration of a crime were all positively associated with victimization. Approximately half of the reported personal crimes were disclosed to a health care professional but only in 16% of the cases the involved psychiatrist report to know about the incident.

**Conclusion:**

This study confirms that people with a history of psychosis have an increased risk of becoming the victim of a crime. Although our results suggest that in fifty percent of cases the patients did share the information with professionals, a substantial proportion of incidents appear to go still unnoticed.

## Introduction

Approximately one in five people with a psychotic disorder report to have been victimized in the preceding year [1,2], which is four to six times higher than the prevalence rate in the general population [1,3]. Victimization is defined as an event in which an individual is the target of a criminal act [4]. With an apparent necessity to address the increased victimization risk and its consequences for treatment, professionals are faced with the problem that patients not always share their experience(s) with them.

A small number of studies that directly address the specific question of whether patients with a psychotic disorder disclose their victimization, suggests disclosure rates are to be low. A study conducted in 1999, involving 234 patients in mental healthcare, found that only 16% of the male and 18% of the female participants disclosed their most traumatic victimization experience to their service provider [5]. Compared to community controls, patients with a psychotic disorder are also less likely (OR 0.50) to have a police record of their victimization incident [6].

Deciding not to disclose victimization, with disclosure here referring to the act of telling someone about victimization experience(s) regardless of whether it is officially recorded [7], can be made because of several reasons. Patients may feel that they are to blame [8], feel ashamed or be afraid that the abuser will find out [9], or expect that others will not believe or help them [5]. In psychosis specifically, Short et al. [6] furthermore suggests that clinical factors such as paranoia or persecutory delusions could prevent people from reporting their experience(s) to an authority figure. Non-disclosure is even more likely when the offender is familiar to the victim [5]. The victim may want to protect the offender or does not consider the violence as victimization [10]. With half [11] to two thirds [12] of the perpetrators being someone familiar, making the registered cases likely to be only the tip of the iceberg.

In urging clinicians to pay close attention to patients’ possible victimization experience, we would like to briefly touch upon some common concerns that can be empirically allayed. It is possible that clinicians are reluctant to address the topic due to the fear that talking about traumatic experiences could disturb or harm the patient [13,14]. This fear is groundless as treating trauma in psychosis has shown to be safe and effective [15,16]. Another reason not to address victimization is fear of false allegations due to psychotic symptoms [14]. A review on retrospective reports however showed that false reports were rare and that under-reporting was common [17]. Whereas self-reports are reasonably stable over time and are not associated with current severity of psychotic symptoms [18]. In addition, although there is some information available on prevalence and risk factors, results vary widely between studies [19] and little is known about the context of the incidents. Who are the perpetrators and where does the victimization take place?

The aim of the current study is fivefold: (1) To explore the prevalence of victimization in patients with a psychotic disorder and to compare this with the relative prevalence rates in the general population in the same region; (2) to describe relevant contextual factors in these patients, such as type of crime, location of the crime, and whether or not the perpetrator was known to the victim; (3) to learn more about disclosure, that is whether and to whom patients disclose the fact that they were victimized; (4) to examine the psychiatrist’s awareness of victimization of their patient; (5) to explore the association of sociodemographic and clinical risk factors with victimization (e.g. age, symptoms, substance use).

## Method

### Sample and procedure

For this study, data on victimization was gathered as part of the ongoing Pharmacotherapy monitoring and outcome survey (PHAMOUS), a longitudinal observational cohort study that yearly assesses the physical and mental health of in- and outpatients with a psychotic disorder in four mental healthcare institutions in the Northern and more rural region of the Netherlands. For the PHAMOUS screening all patients who use antipsychotic medication are invited. First, the procedure was explained to the patients. The PHAMOUS screenings are carried out by trained research nurses as part of standard clinical practice. The PHAMOUS protocol was approved by the local ethical committee and conducted in accordance with the guidelines of the Helsinki Declaration. For a more detailed description of the sample and procedures, see Bartels-Velthuis et al. [20]. From December 2010 to September 2011, as part of the so called VICTROM study, a victimization questionnaire was added to the PHAMOUS screening. Patients are given the opportunity to opt out from parts of the protocol, also for the VICTROM screening, and could choose to have their anonymized data removed from the research database [20]. Since the PHAMOUS study has a naturalistic approach as part of the routine outcome assessment to evaluate treatment, no information on patients who did not participate in this assessment was recorded.

### Participants

In this study adult patients with a diagnosis in the psychotic spectrum according to DSM-IV [21] were included in the analyses here presented.

### Instruments

#### Victimization and perpetration of a crime

*Dutch crime and victimization survey (IVM)* (In Dutch: Veiligheidsmonitor) [22] was used to investigate the prevalence of self-reported victimization, the characteristics of the crime and whether the victim disclosed the victimization. The IVM was translated and adapted by the Dutch Ministry of Justice and Security, based on the international self-report crime victimization survey [23]. It is annually administered to people in the general population to assess e.g. feelings of safety, victimization and neighborhood livability.

There is no psychometric information on the IVM but the instrument is recently used in several studies on victimization in people in mental healthcare and no indication of invalidity of response is reported [24,25]. In order to investigate the prevalence of self-reported victimization, we added the following IVM subscales to the PHAMOUS screening: property crimes (burglary, theft, pickpocketing, vandalism), personal crimes (threats, assaults, sexual crimes) and other crimes (e.g. fraud, internet scam). With the help of a timeline and sets of examples, patients were asked whether they had experienced different forms of victimization (yes/no). Additionally, patients provided extra information on which items were stolen. The victimization prevalence rates were calculated for both the last year and the preceding five years. Finally, patients were also asked whether they had committed one or more of these crimes themselves in the previous year. Potential risk factors were calculated based only on the preceding year. Prevalence of victimization in our sample was compared to data from the general population, that was collected in 2011 in the same three Northern provinces in which the four mental health institutions are located [22]. This information is presented to provide some insight in the relative height of the prevalence found.

In order to answer aim 2, questions on the perpetrator and location of the crime were added. For research aim 3, questions were added surrounding disclosure (yes/no) and if applicable: to whom disclosure had taken place. Given the already sizeable PHAMOUS test battery, our assessment procedure had to remain relatively short. As such, these extra questions were restricted to the most recent incident only.

To assess psychiatrists’ awareness of incidents of victimization, the involved psychiatrist was asked to complete a short questionnaire whether, in the previous year, their patient had been a victim of one or more of nine different types of victimization corresponding to the IVM subscales (answer options yes, no or don’t know).

The following socio-demographic and clinical factored were obtained from PHAMOUS in order to explore possible risk factors for victimization.

*Demographic and psycho-social information* was retrieved from the general PHAMOUS screening to explore possible risk factors for victimization. Information on age, gender, in- or outpatient status and substance use (‘do you use alcohol yes/no, do you use drugs yes/no), diagnosis (DSM-IV) and comorbid diagnosis (yes/no) were included. To assess other patient characteristics, information from the following questionnaires was used:

The *Positive and Negative Syndrome Scale Remission* (PANSS-R) was administered to assess the severity of the psychiatric symptoms in eight items [26]. Positive symptoms were assessed with the items P1 ‘delusions’, P2 ‘conceptual disorganization’, and P3 ‘hallucinations’, negative symptoms with the items N1 ‘blunted affect’, N4 ‘passive/apathetic social withdrawal’, and N6 ‘lack of spontaneity and flow of conversation’, and general symptoms with the items G5 ‘mannerisms and posturing’ and G9 ‘unusual thought content’.

The *Health of the Nation Outcome Scale (HoNOS)* [27] measures mental health and social functioning of people with a mental illness and consists of twelve items covering four subscales (behavior, impairment, symptoms and social problems). The items are rated on a 5-point scale ranging from 0 = no problem to 4 = severe to very severe problems.

The *Global Assessment of Functioning scale (GAF)* was used to measure psychological, social, and occupational functioning (APA, 1994). In this study, the adapted version of the GAF was used with separated ratings for symptoms and disability [28,29]. The scale ranges from 1 to 100, with higher scores reflecting less symptoms and a better level of functioning.

The *Manchester Short Assessment of Quality of Life* (MANSA) [30] assesses quality of life (QoL), with twelve items evaluating QoL (score 1–7) on several psychosocial domains (e.g. financial situation, employment, physical health, mental health) and includes also four questions about violence and social interactions (answer options yes/no). The mean scores of the twelve items and the dichotomous social interaction scores were used in the analyses. As the questions on violence (victim or perpetrator) strongly resemble questions of the IVM, these outcomes are given separately and are used to explore their association with the IVM personal crime and perpetration score.

### Analyses

The association between personal crime score and the perpetration score of the IVM and the equivalent MANSA questions was calculated with the Pearson correlation coefficient. To examine the univariate relation between patient characteristics and whether or not an incidence was disclosed, an independent sample t-test was used for continuous variables and chi-square or Fisher’s exact test for categorical variables. Association between both property and personal victimization and patient characteristics, were analyzed with logistic regression. Variables that were significant at p<0.05 level were included into a multivariate model. Backward stepwise logistic regression was performed to iteratively remove the least useful predictor one at a time, and identify the model that is best associated with property or personal victimization. Analyses were conducted with the SPSS package for IMB statistics version 24.0.

### Ethical standards

The authors assert that all procedures contributing to this work comply with the ethical standards of the relevant national and institutional committees on human experimentation and with the Helsinki Declaration of 1975, as revised in 2008, and is approved by the local ethical committee of the University Medical Center of Groningen.

## Results

In total 42.8% of all patients with a diagnosis in the psychosis spectrum in care at four mental healthcare institutions participated in the PHAMOUS study in the year VICTROM was added [31]. Of this group 65.9% (n = 581) completed the victimization survey during the 9-months study period. As patients may opt to have their data deleted from the databank, specifics surrounding non-response are not available. In total 353 patients were included in this study. Their mean age was 49.5 years (sd = 11.0) and 35% was female. All patients were 18 years or older and 101 (29%) patients were diagnosed with a comorbid disorder (e.g. substance dependence, mood disorder, personality disorder) according to DSM-IV.

### Prevalence rate

The IVM results in Table 1 show that in the preceding year 18.8% of the patients reported to have been victim of a property crime and 9.7% reported a personal crime, including 1.8% sexual victimization (for females only this is 4.1%). In the preceding five years, property crime was 32.9% and personal crime was 20.4%. Compared to the general population in the same region (N = 9135), the annual prevalence rates for patients were higher for all specific victimization types except vandalism (9.3% in the population versus 6.6% in patients). On the MANSA-questions, 5.6% of the patients reported to have been the victim of violence in the preceding year, which correlates 0.23 (p = < 0.001) with the IVM personal crime scale.

**Table 1:** Victimization prevalence rate for the general population and for patients with a psychotic disorder.

	One year prevalence rate general population	One year prevalence rate patients		Five year prevalence rate patients	
Type of victimization	N = 9135 (%)^2^	%	n/N	%	n/N
IVM Property Crime	11.3	18.8	62/329	32.9	110/334
Attempted burglary	1.1	3.8	13/340	6.8	23/340
Burglary	1.3	6.8	23/340	10.9	37/340
Theft bicycle	4.1	6.8	20/343	12.8	44/343
Theft other	4.4	5.8	20/343	11.1	38/343
Pick-pocketing	1.2	1.2	4/342	3.2	11/342
Robbery	0.1	0.0	0/338	0.6	2/338
IVM Vandalism	9.3	6.6	23/349	10.9	38/349
IVM Personal crime	4.5	9.7	34/334	20.4	68/334
Sexual harassment/assault	1.5	1.8	6/343	5.0	17/343
Threats of violence	3.0	6.5	22/336	13.1	44/336
Physical assault	0.7	3.6	12/335	7.2	24/335
IVM Crime (other)^1^	0.8	4.1	14/340	7.9	27/342
IVM Total of all crimes	21.3	32.6	105/322	51.8	170/328
MANSA victim of violence		5.6	17/306		
IVM perpetrator		5.6	19/338		
MANSA accused of a crime		2.9	9/307		

^1^ For example stalking, internet scam, fraud, psychological abuse.

^2^ Prevalence rates for people in same region and in the same year as the study group (Veiligheidsmonitor, 2011)

In addition, post-hoc analyses showed that 22% of the property crime victims also reported being victim of a personal crime, and 50% of the personal crime victims reported being victim of property crime. Reporting one crime was significantly associated with reporting another crime (OR = 4.24, CI95% = 2.00–8.96, p = <0.001).

5.6% of the patients reported that they had been perpetrator of a personal crime in the preceding year on the IVM, including sexual harassment or assault (1.2%), threats of violence (3.3%), physical assault (2.1%) and other crimes (2.1%). Only 2.9% of patients reported that they were accused of a crime in the MANSA item, which correlates 0.21 (p = <0.001) with the IVM perpetration scale.

### Characteristics of the victimization and its context

Most patients reported that the last time they experienced sexual harassment or assault the perpetrator was a ‘friend’ or acquaintance (62.5%). The same holds for threats of violence (34.1%) and physical assault (36.4%), only the latter was also frequently committed by a fellow patient (36.4%). Strangers were most often responsible for crimes in the ‘other’ category (44%). Family members and (ex) partners were least often reported as perpetrators for all victimization types. Sexual harassment or assault most often occurred in the victim’s home (45.7%) or other familiar places (26.7%); in 20% of cases this occurred in the perpetrator’s home. Threats and physical assault happened mostly in a public place or in the victim’s home. Other crimes could happen at any location (see Table 2).

**Table 2:** Perpetrators and locations of the crime based on the IVM.

		Perpetrator of the crime^1^ % (n)				
Type of crime	N	(Ex)partner	Family member	Friend/ acquaintance	Fellow patient	Stranger
Sexual harassment or assault	16	12.5 (2)	0 (0)	62.5 (10)	18.8 (3)	6.3 (1)
Threats of violence	44	6.8 (3)	4.5 (2)	34.1 (15)	22.7 (10)	31.8 (14)
Physical assault	22	0 (0)	0 (0)	36.4 (8)	36.4 (8)	27.3 (6)
Crime (other)^2^	25	12.0 (3)	4.0 (1)	28.0 (7)	12.0 (3)	44.0 (11)
		Location of the crime^1^ % (n)				
Type of crime	N	Public place	Own home	Familiar home/place^3^	Home of the offender	Other^4^
Sexual harassment or assault	15	6.7 (1)	45.7 (7)	26.7 (4)	20.0 (3)	0 (0)
Threats of violence	42	38.1 (16)	40.5 (17)	11.9 (5)	2.4 (1)	7.1 (3)
Physical assault	23	52.2 (12)	34.8 (8)	8.7 (2)	0 (0)	4.3 (1)
Crime (other)^2^	25	36.0 (9)	40.0 (10)	16.0 (4)	0 (0)	8.0 (2)

^1^ Last time the victimization happened in the preceding five years

^2^ For example stalking, internet scam, fraud, psychological abuse.

^3^ Home of a friend, family member, acquaintance or at work

^4^ Internet, on the phone, in a letter or other

On the question which item was stolen, participants mentioned in total 51 different items, with the most common items being clothing (12 times), money/wallet/debit card (11 times), CDs (5 times) or a mobile phone (4 times). Other items that were mentioned were cigarettes, a lighter, plants, keys, a magazine, a camera, or a computer screen. A post-hoc analysis showed that 10.4% of the inpatients was victim of burglary versus 4.6% of the outpatients (OR = 2.55 CI = 0.89–7.34, p = 0.082).

### Disclosure and awareness

Of the patients who reported sexual harassment or assault in the preceding five years, 62.5% disclosed the most recent incident to a health care professional and 18.8% reported it to the police. Threats of violence were about equally disclosed to a non-professional (45.5%) as to a health care professional (38.6%). The same holds true for incidents of physical assault (43.5% and 43.5%). In the case of other crimes people most often disclosed to a non-professional (66.7%). On average, including all victimization types, 11% of the patients did not report the incident to anyone. No relationship was found between patient characteristics and disclosure of threats and (sexual) violence to a professional or non-professional (see Table 3).

**Table 3:** Disclosure of victimization and awareness of the psychiatrist.

		**Disclosure**^**1**^ **% (n)**			
Type of victimization	N	health care professional	police	Non- professional^2^	no
Sexual harassment or assault	16	62.5 (10)	18.8 (3)	50.0 (8)	6.3 (1)
Threats of violence	44	38.6 (17)	27.3 (12)	45.5 (20)	20.5 (9)
Physical assault	23	43.5 (10)	39.1 (9)	43.5 (10)	8.7 (2)
Crime (other)	24	54.2 (13)	33.3 (8)	66.7 (16)	8.3 (2)
	**Disclosure**^**1**^ **of personal crime**^**3**^ **% (n)**				
Characteristic	professional^4^	no or non-professional^2^		X^2^/t (df)	*p* (two-sided)
Age^5^ mean (sd)	46.58 (10.48)	44.50 (11.15)		-0.75 (65)	0.67
Gender^6^ male	63.3 (31)	36.7 (18)		0.99 (1)	0.32
female	76.5 (13)	23.5 (4)			
Inpatient/sheltered housing^7^	81.8 (9)	18.2 (2)		(1)	0.46
Outpatient	63.9 (23)	36.1 (13)			
Type of victimization	victim^8^ (n)	**Awareness psychiatrist**^**8**^ **% (n)**			
		yes		no	does not know
**Property Crime**					
Total	yes (63)	12.7 (8)		60.3 (38)	27.0 (17)
	no (1111)	1.0 (11)		78.5 (872)	20.5 (228)
(Attempted) burglary	yes (8)	12.5 (1)		62.5 (5)	25.0 (2)
	no (224)	0.9 (2)		(177)	20.1 (45)
Theft	yes (36)	16.7 (6)		61.1 (22)	22.2 (8)
	no (197)	2.0 (4)		(142)	25.9 (51)
Vandalism	yes (16)	6.3 (1)		56.2 (9)	37.5 (6)
	no (226)	1.3 (3)		(177)	20.4 (46)
Pick-pocketing	yes (3)	0.0 (0)		66.7 (2)	33.3 (1)
	no (232)	0.4 (1)		(187)	19.0 (44)
Robbery	yes (0)	0.0 (0)		0.0 (0)	0 (0)
	no (232)	0.4 (1)		81.5 (189)	18.1 (42)
**Personal crime**					
Total	yes (31)	16.1 (5)		64.5 (20)	19.4 (6)
	no (663)	1.2 (8)		73.6 (488)	25.2 (167)
Sexual harassment or assault	yes (3)	33.3 (1)		0.0 (0)	66.7 (2)
	no (232)	0.4 (1)		(169)	26.7 (62)
Threats of violence	yes (19)	10.5 (2)		68.4 (13)	21.1 (4)
	no (210)	1.9 (4)		(149)	27.1(57)
Physical assault	yes (9)	22.2 (2)		77.8 (7)	0 (0)
	no (221)	1.4 (3)		76.9 (170)	21.7 (48)
**Crime (other)**	yes (6)	33.3 (2)		66.7 (4)	0 (0)
	no (228)	0.4 (1)		75.0 (171)	24.6 (56)

^1^ Last time the victimization happened in the previous five years

^2^ Partner, family member, friend, acquaintance

^3^ Including sexual harassment or assault, threats of violence and physical assault

^4^ Health care professional and/or police

^5^ Independent Sample t-test

^6^ Chi Square Test

^7^ Fisher’s Exact Test

^8^ In the previous year

Table 3 also presents the psychiatrist’s awareness of the victimization of their patient in the preceding year. When patients reported to have been victim of a personal crime (31 times), the psychiatrist most often (65%) reported that their patient was not victimized. In 16% of the cases they were aware and in 19% of the cases they noted not to know. Results were similar for property crimes. In case the patient reported no victimization, the psychiatrist most often agreed by responding with no victimization. In a total of 20 cases (57%), the psychiatrists thought that their patient was victimized but the patient reported no victimization.

### Factors associated with victimization

Table 4 presents the univariate analyses and Table 5 depicts the multivariate analyses of potential risk factors. Property crime was on univariate level associated with the age group of 36–45 years, drug use, being a perpetrator, higher score on the behavioral problems subscale of the HoNOS and lower quality of life. In the multivariate analyses, all these associations remained significant at p<0.05, except for drug use. A multicollinearity check revealed that the variance of the regression coefficient of drug use was dependent on the variance coefficient of perpetrator and the HoNOS behavior scale, indicating collinearity. In the second step of the multivariate analyses only perpetration (p = 0.046) and the HoNOS behavior scale (p = 0.046) were significantly positively related to property crime. The MANSA mean score (p = 0.003) was significantly (negatively) related to property crime. The goodness of fit for the property crime model was good for model 1 (X^2(5)^ = 23.06, R^2^ = 0.120, p = <0.001) and model 2 (X^2 (3)^ = 21.47, R^2^ = 0.112, p = <0.001).

**Table 4:** Univariate analyses of factors associated with personal and property crime.

	Victim of a property crime				Victim of a personal crime			
	N	Yes victim n (%)	No victim n (%)	Odds Ratio (95% CI)	N	Yes victim n (%)	No victim n (%)	Odds Ratio (95% CI)
Gender male	215	55 (25.6)	160 (74.4)	1.41 (0.81–2.46)	219	26 (11.9)	193 (88.1)	2.02 (0.85–4.81)
female	112	22 (19.6)	90 (80.4)	1	112	7 (6.3)	105 (93.8)	1
Age 25–35	39	10 (25.6)	29 (74.4)	1.38 (0.62–3.06)	40	8 (20.0)	32 (80.0)	4.10 (1.56–10.82)**
36–45	86	27 (31.4)	59 (68.6)	1.83 (1.04–3.24)*	85	14 (16.5)	71 (83.5)	3.24 (1.43–7.33)**
46+	205	41 (20.0)	164 (80.0)	1	209	12 (5.7)	197 (94.3)	1
Inpatient/sheltered housing	65	15 (23.1)	50 (76.9)	0.94 (0.48–1.83)	68	6 (8.8)	62 (91.2)	0.86 (0.33–2.27)
Outpatient	177	43 (24.3)	134 (75.7)	1	178	18 (10.1)	160 (89.9)	1
Alcohol yes	107	31 (24.4)	96 (75.6)	1.13 (0.67–1.92)	131	11 (8.4)	120 (91.6)	0.93 (0.42–2.05)
no	189	42 (22.2)	147 (77.8)	1	189	17 (9.0)	172 (91.0)	1
Drugs yes	32	13 (40.6)	19 (59.4)	2.62 (1.22–5.61)*	33	9 (27.3)	24 (72.7)	5.03 (2.06–12.25)***
no	285	59 (20.7)	226 (79.3)	1	288	20 (9.0)	268 (93.1)	1
Perpetrator yes	18	9 (50.00)	9 (50.00)	3.52 (1.34–9.20)**	19	9 (47.4)	10 (52.6)	10.33(3.84–27.78)***
no	307	68 (22.1)	239 (77.9)	1	306	25 (8.0)	287 (92.0)	1
Comorbid disorder yes	94	23 (24.5)	71 (75.5)	1.07 (0.61–1.86)	97	15 (15.5)	82 (84.5)	2.10 (1.02–4.33)*
no	236	55 (23.3)	181 (76.7)	1	237	19 (8.0)	218 (92.0)	1
PANSS Mean (sd) Total	287	1.99 (0.63)	2.02 (0.84)	0.95 (0.67–1.35)	296	1.97 (0.66)	2.02 (0.82)	0.94 (0.58–1.52)
Positive symptoms	287	2.09 (0.83)	1.92 (1.01)	1.19 (0.91–1.57)	296	2.04 (0.83)	1.95 (0.99)	1.10 (0.76–1.60)
Negative symptoms	287	2.04 (0.88)	2.28 (1.08)	0.79 (0.60–1.05)	296	2.14 (0.89)	2.24 (1.07)	0.91 (0.63–1.32)
General symptoms	269	1.71 (0.76)	1.72 (0.92)	0.98 (0.72–1.35)	276	1.59 (0.72)	1.72 (0.90)	0.83 (0.51–1.35)
HoNOS Mean (sd) Behavior	286	1.40 (2.01)	0.76 (1.10)	2.37 (1.35–4.16)**	291	0.53 (0.73)	0.28 (0.43)	2.24 (1.18–4.27)*
Impairment	286	1.94 (1.83)	1.97 (1.72)	0.99 (0.72–1.36)	291	0.91 (0.81)	0.99 (0.87)	0.90 (0.57–1.42)
Symptoms	286	3.15 (2.48)	3.30 (2.45)	0.99 (0.71–1.38)	291	1.16 (0.82)	1.10 (0.83)	1.08 (0.68–1.70)
Social problems	286	3.65 (2.85)	3.42 (2.53)	1.16 (0.76–1.75)	291	0.93 (0.72)	0.87 (0.65)	1.14 (0.64–2.02)
GAF Mean (sd) Disability	215	56.65 (11.93)	56.31 (13.18)	1.00 (0.98–1.03)	218	56.29 (13.94)	56.06 (12.96)	1.00 (0.97–1.04)
Symptoms	233	55.48 (12.74)	54.93 (14.82)	1.00 (0.98–1.02)	238	51.92 (16.88)	54.70 (13.99)	0.99 (0.96–1.02)
MANSA* Mean (sd)	293	4.70 (0.89)	5.09 (0.84)	0.60 (0.44–0.82)**	296	4.74 (1.12)	5.04 (0.81)	0.67 (0.43–1.03)
Having a good friend yes	198	49 (24.7)	149 (75.3)	1.26 (0.69–2.30)	200	18 (9.0)	182 (91.0)	0.74 (0.33–1.63)
no	92	19 (20.7)	73 (79.3)	1	93	11 (11.8)	82 (88.2)	1
Spoken with a friend past week yes	176	41 (23.3)	135 (76.7)	0.97 (0.56–1.69)	179	14 (7.8)	165 (92.2)	0.55 (0.26–1.20)
no	113	27 (23.9)	86 (76.1)	1	113	15 (13.3)	98 (86.7)	1

*p ≤ 0.05

**p < 0.01

***p < 0.001

**Table 5:** Multivariate analyses of factors associated with property crime and personal crime.

	Step 1		Step 2	
	β (SE)	Odds Ratio (95% CI)	β (SE)	Odds Ratio (95% CI)
**Property crime**^**1**^				
Perpetrator yes	1.04 (0.54)	2.82 (0.98–8.08)*!	1.07 (0.54)	2.93 (1.02–8.41)*
HoNOS behavior	0.56 (0.29)	1.76 (0.99–3.12)	0.59 (0.29)	1.80 (1.01–3.21)*
MANSA	-0.51 (0.18)	0.60 (0.42–0.85)**	-0.52 (0.18)	0.59 (0.42–0.84)**
Age 36–45	0.43 (0.33)	1.53 (0.80–2.94)		
Constant	0.91 (0.92)	2.47	1.08 (0.90)	2.95
**Personal crime**^**2**^				
Perpetrator yes	2.34 (0.58)	10.41 (3.34–32.38)***		
Age 25–35	1.63 (0.59)	5.09 (1.62–16.00)**		
36–45	1.14 (0.49)	3.13 (1.20–8.16)*		
Comorbid disorder yes	1.08 (0.47)	2.93 (1.24–6.95)*		
HoNOS behavior	0.65 (0.37)	2.02 (1.00–4.12)*		
Constant	-3.83 (0.47)	0.02***		

^1^Property crime

Step 1 Omnibus tests Step p <0.001, Model p <0.001, Hosmer and Lemeshow p 0.291, Nagelkerke R^2^ = 0.120

Step 2 Omnibus tests Step p 0.451, Model p <0.001, Hosmer and Lemeshow p 0.736, Nagelkerke R^2^ = 0.112

^2^Personal crime

Step 1 Omnibus tests Step p <0.001, Model p <0.001, Hosmer and Lemeshow p 0.116, Nagelkerke R^2^ = 0.243

*p ≤ 0.05

**p < 0.01

***p < 0.001

The univariate and multivariate analyses on personal crime showed a significant association with perpetration (p = <0.001), age group 25–35 (p = 0.005), age group 36–45 (p = 0.019), having a comorbid disorder (p = 0.015) and possibly a higher score on the HoNOS behavior scale (p = 0.051). Drug use was also significant on univariate level, but was excluded from the multivariate analyses due to its dependency on perpetration and the HoNOS behavior scale. The goodness of fit for the final personal crime model was good (X^2(5)^ = 35.79, R = 0.243, p = < 0.001).

## Discussion

### Prevalence rate

Our results support the notion that patients with a psychotic disorder are more vulnerable to victimization than the general public. Approximately one out of ten patients with a psychotic disorder reported to have been victim of a personal crime in the preceding year. Compared to other studies with people with a psychotic disorder [19] who reported rates around 20%, this prevalence rate is relatively low. It is however, still about two times higher than victimization rate in the general population living in the same area as the participant, which was 4,5%. Reports on sexual harassment or sexual assault are particularly rare when comparing our finding of two percent to other studies who found an annual prevalence ranging from thirteen to 51 percent [19]. Differences in sample characteristics and/or methodology may explain part of the lower prevalence rate. For example, compared to other studies [32–34], we included fewer females and the mean age of the patients (49.5 years), which may have decreased the risk of sexual victimization. Another explanation for the lower personal crime rate is that it is a reflection of the lower general crime rate in this rural, Northern part of the Netherlands.

Although the general property crime is Northern Netherlands is relative low (11.3%), we found high prevalence rate of 19% in our sample, which is similar to other studies on people with a psychotic disorder [12,33]. A post-hoc analysis revealed that people living in a mental health facility or sheltered housing reported more burglaries (trend score) than people who received outpatient treatment. In mental health facilities, people often share the same space and have easy access to one another’s rooms, making theft of personal belongings more likely. Vandalism is, contrary to other forms of property crime, less often reported by patients than in the general population [24], due to a higher rate of car vandalisms and car ownership in the last group. In the ‘other’ crime category, consisting of, for example, cyber-crimes, prevalence was higher for patients (4.1%) compared to the general population (1.1%), indicating a possible vulnerability in this area.

### Characteristics of the victimization and its context

People with a psychotic disorder are often victimized by someone they know. The high involvement of the patients’ acquaintances in violent threats, physical assaults and sexual victimization, that is in more than two third of the cases is in line with other research in people with psychosis [12,33]. In the general population in the Netherlands in 2011, approximately half of these offences were committed by someone familiar.

Even more salient is the finding that in between one out of five to one out of three cases, a fellow patient was responsible for the crime. De Mooij et al. [12] found similar results and suggested that inpatients may have more conflicts as they live closely together, resulting in less privacy and more irritation toward each other.

Most sexual crimes and threats of violence occurred in the victim’s home, a familiar home/place or the offender’s home. These findings are the more worrying, since victimization often leads to adverse outcomes when the victimization takes place in a ‘safe’ location where safety perception is high [35,36]. The results suggest that including the social network and improvement of social skills and social cognition may be important in the prevention of victimization. For example, patients may miss important social cues, are possibly seen as an ‘easy’ target and are in contact with potential offenders [19] making victimization more likely. Additionally, it is also advised to increase the awareness of the high risk of victimization in inpatient setting.

### Disclosure and awareness

Whereas Marley & Buila [5], reporting rates of disclosure of 16% (men) and 18% (women) in our study approximately half of the patients disclosed the fact that they were victimized to a health care professional. Methodological differences make direct comparison difficult. Possibly the awareness of professionals is increasing over time and this report can be seen as an attempt to update from the studies of before 2000. In contrast to these relative high disclosure percentages, psychiatrists in general were not well-informed where incidences of violence were concerned. Of all the cases in which a patient reported victimization, only 16% of the responsible psychiatrist reported to be aware. These outcomes could be a result of non-disclosure by patients, but also because of differences in the examined time frame or infrequent contact between psychiatrist and the patient, as care is often shared with case managers and psychologists. The lack of knowledge of the psychiatrists is in line with a recent study by Sampson & Read [37] who compared the recorded incidents of victimization in the files of service users in 1997 with recent files (after 2002). Although they found an increase in recordings, the number was still below the expected victimization prevalence rate, especially when the service user was male and/or had a psychotic disorder.

Whether due to unawareness or nondisclosure, all in all, the fact that professionals were not always aware of incidents indicates that victimization is typically not addressed by psychiatrists. Given the severity of some of the incidents (e.g. physical and sexual violence), such unawareness may have important ramifications for the patient in terms of treatment.

### Possible risk factors for victimization

#### Property crime

Being the victim of a property crime is associated with being a perpetrator oneself, and problems with alcohol and drug use and aggressive, disruptive or agitated behavior. Other studies have also found relationships between violent victimization and risky behaviors such as substance abuse and violent behavior [19,38]. It may be possible, however, that these relationships are found due to common underlying risk factors. For example, substance use is associated with being a perpetrator of a crime [39] and at the same time brings people in close proximity to possible offenders (e.g. drug dealers, addicts). The association found between a lower quality of life and property crime may also be explained by shared underlying factors, in this case environmental factors such as living situation. People living in sheltered housing or in an inpatient setting reported more burglaries and have also been found to experience on average a lower quality of life [40].

#### Personal crime

Similar to property crime, being a perpetrator of a crime and a higher score on the HoNOS behavior scale were associated with a higher prevalence of personal crimes. These findings are in line with a meta-analysis [19] showing a significant association between being a perpetrator of a crime, substance use and victimization, and suggested that problematic behavior due to the psychiatric problems and/or a restriction in social functioning is associated with exposure to high risk situations [19]. In contrast, no such association was found for a single question on alcohol use. One likely explanation for this finding may be found in the dichotomous nature of the question (yes/no) rather than a question whether the alcohol use was problematic and to what extent.

In line with research in the general population [41], adolescents and young adults reported violent incidents significantly more often. Finally, having a comorbid diagnosis was also associated with victimization of a personal crime. Again, this is not surprising as this group in general has more severe persistent substance abuse problems [42] and more often shows violent behavior [43], indicating that this group is more susceptible to victimization.

Our results suggest that property crime and personal crime share some of the same risk factors. This possibly explains why people who were victim of a personal crime had four times the odds of experiencing property crime. This so-called poly-victimization is common in people with a severe mental illness [24].

Where other research suggests a relationship between symptoms and victimization, especially for hallucinations and delusions [19], in this study no such association was found. This may be due to the fact that the PANSS scores were relatively low and most people at the time of the interview did not experience positive symptoms. Another reason is that the time between the victimization and symptoms assessment is unknown and could be as long as 12 months. Exploration of possible risk factors shows that some patients are more vulnerable to victimization than others. Psychiatrists should be aware of the increased victimization risk of patients who show risky behavior such as substance abuse or aggressive behavior.

### Limitations and future research

A number of limitations to our study should be noted. First, the cross-sectional design of the study hinders drawing conclusions about causation. In future research, a longitudinal or prospective design is preferred. Second, the explained variance of the models was low, suggesting other risk factors might also predict victimization, such as living in a disadvantaged neighborhood or problems in social cognitions [19]. Third, we only asked whether people had disclosed their most recent incident. We have no information on how disturbing this incident was and whether there was a need for disclosure. Finally, the lack of specific postal codes led to a more general comparison with the whole population in the region and prevented us from making neighborhood-matched comparisons.

To measure victimization in the future it is recommended to use a detailed questionnaire such as the IVM, as this may elicit a better recall of incidents as it has specific as it has specific victimization questions. More research is necessary to investigate trajectories of victimization, overlap with criminal behavior, and detect potential subgroups with an even higher risk for victimization.

### Conclusion

Our findings of high prevalence of victimization among persons with a psychotic disorder are in line with earlier studies. However, we have additionally found that some serious incidents may still go unnoticed, which possibly hampers rehabilitation and the prevention of revictimization. Our findings further highlight that offenders are only rarely outsiders, most often belonging to the victim’s own social network, possibly as a result from risky behaviors and the restriction of shared living situations in inpatient settings

These results suggest that psychiatrists should discuss victimization more often with their patients, especially when they show risky behavior such as substance abuse or aggressive behavior. There may be strategies developed and implemented in mental health care that increase the awareness of risks, and thus prevent victimization[24]. Further prevention should take the form of interventions focused on improvement of social cognition and skills-based training that increases awareness and tools to respond to threatening situations [44].
